# Bacterial Enrichment Cultures Biotransform the Mycotoxin Deoxynivalenol into a Novel Metabolite Toxic to Plant and Porcine Cells

**DOI:** 10.3390/toxins13080552

**Published:** 2021-08-09

**Authors:** Ilse Vanhoutte, Caroline De Tender, Kristel Demeyere, Mohamed F. Abdallah, Sarah Ommeslag, Pieter Vermeir, Sarah De Saeger, Jane Debode, Evelyne Meyer, Siska Croubels, Kris Audenaert, Leen De Gelder

**Affiliations:** 1 Laboratory of Environmental Biotechnology, Department of Applied Biosciences, Faculty of Bioscience Engineering, Ghent University, 9000 Ghent, Belgium; Ilse.Vanhoutte@UGent.be; 2 Plant Sciences Unit, Flanders Research Institute for Agriculture, Fisheries and Food (ILVO), 9820 Merelbeke, Belgium; caroline.detender@ilvo.vlaanderen.be (C.D.T.); Sarah.Ommeslag@ilvo.vlaanderen.be (S.O.); Jane.Debode@ilvo.vlaanderen.be (J.D.); 3 Computer Science and Statistics, Department of Applied Mathematics, Faculty of Sciences, Ghent University, 9000 Ghent, Belgium; 4 Department of Pharmacology, Toxicology and Biochemistry, Faculty of Veterinary Medicine, Ghent University, 9820 Merelbeke, Belgium; Kristel.Demeyere@UGent.be (K.D.); Evelyne.Meyer@UGent.be (E.M.); Siska.Croubels@UGent.be (S.C.); 5 Centre of Excellence in Mycotoxicology and Public Health, Department of Bioanalysis, Faculty of Pharmaceutical Sciences, Ghent University, 9000 Ghent, Belgium; Mohamed.Fathi@UGent.be (M.F.A.); Sarah.DeSaeger@UGent.be (S.D.S.); 6 Laboratory of Chemical Analysis (LCA), Department of Green Chemistry and Technology, Faculty of Bioscience Engineering, Ghent University, 9000 Ghent, Belgium; Pieter.Vermeir@UGent.be; 7 Laboratory of Applied Mycology and Phenomics, Department of Plants and Crops, Faculty of Bioscience Engineering, Ghent University, 9000 Ghent, Belgium; Kris.Audenaert@UGent.be

**Keywords:** deoxynivalenol derivatives, enrichment, biotransformation, bioassay, cytotoxicity assay, metabarcoding

## Abstract

The mycotoxin deoxynivalenol (DON), produced in wheat, barley and maize by *Fusarium graminearum* and *Fusarium culmorum*, is threatening the health of humans and animals. With its worldwide high incidence in food and feed, mitigation strategies are needed to detoxify DON, maintaining the nutritional value and palatability of decontaminated commodities. A promising technique is biological degradation, where microorganisms are used to biotransform mycotoxins into less toxic metabolites. In this study, bacterial enrichment cultures were screened for their DON detoxification potential, where DON and its potential derivatives were monitored. The residual phytotoxicity was determined through a bioassay using the aquatic plant *Lemna minor* L. Two bacterial enrichment cultures were found to biotransform DON into a still highly toxic metabolite for plants. Furthermore, a cytotoxic effect was observed on the cellular viability of intestinal porcine epithelial cells. Through liquid chromatography high-resolution mass spectrometry analysis, an unknown compound was detected, and tentatively characterized with a molecular weight of 30.0 Da (i.e., CH_2_O) higher than DON. Metabarcoding of the subsequently enriched bacterial communities revealed a shift towards the genera *Sphingopyxis*, *Pseudoxanthomonas*, *Ochrobactrum* and *Pseudarthrobacter*. This work describes the discovery of a novel bacterial DON-derived metabolite, toxic to plant and porcine cells.

## 1. Introduction

The mycotoxin deoxynivalenol (DON) is produced in wheat, barley and maize mainly by *Fusarium graminearum* and *Fusarium culmorum* in the field. DON belongs to the trichothecenes type B, a group of secondary fungal metabolites which are harmful to human and animal health. They are non-volatile, low molecular weight (MW) sesquiterpene epoxides and usually contain an epoxide at C-12 and -13, which is essential for their toxicity [1]. DON interferes with the active site of peptidyl transferase on ribosomes and thus inhibits the elongation step of protein synthesis in eukaryotes [1]. The molecule induces a ribotoxic stress, leading to the activation of mitogen-activated protein (MAP) kinases, cell cycle arrest and apoptosis [2]. Its toxicological effects mainly concern the immune system and the gastrointestinal tract [3]. Chronic DON ingestion at low doses is associated with decreased weight gain, anorexia, decreased nutritional efficiency and altered immune function, with apparent species differences. Feed intake was significantly suppressed in pigs at naturally contaminated DON levels starting from 0.95 mg/kg [4]. Decreased body weight was observed in pigs fed with 3.5 mg/kg DON, where the no-observed adverse effect level (NOAEL) was estimated at 30 µg/kg body weight/day [5]. Acute higher doses provoke nausea, diarrhea and vomiting [6]. DON is of particular importance due to its frequent occurrence in toxicologically relevant concentrations [7] and was found to be the most prevalent mycotoxin, with 55% of the samples testing positive in feed collected during an 8-year period worldwide [8,9]. The European Commission [10] has imposed maximum levels for DON in food (such as 1.25 mg/kg in unprocessed grains) and guidance values for DON in feeding stuffs [11].

In addition to severe human and animal health problems, mycotoxins cause major economic losses at all levels of food and feed production, including crop and animal production, and crop distribution and processing [12]. Treatments are needed either to reduce mycotoxin concentrations in food and feed or to alleviate their adverse effects on animals and humans. First of all, strategies for pre-harvest prevention are important to implement, such as appropriate field management and optimal storage practices [13]. The use of resistant cultivars, fungicide use and application of biological control agents also promises prevention or intervention techniques to reduce the amount of mycotoxins [14,15]. Further, post-harvest detoxification strategies are needed to remediate mycotoxin-contaminated crops after harvest. These strategies consist of chemical, physical or biological methods. In the feed industry, the use of binders is most widespread. These are adsorption agents that suppress or reduce the absorption of mycotoxins in the gastrointestinal tract and promote the excretion of mycotoxins [16]. However, their efficacy in reducing mycotoxin contamination is variable, and most of the commercial binding agents present insufficient effect against DON [17]. Therefore, biological detoxification methods, that biotransform mycotoxins into less toxic metabolites, are generally more specific, efficient and environmentally friendly. These agents maintain the nutritional value and palatability of decontaminated commodities [18].

To obtain DON-biotransforming organisms, enrichment is commonly used. Starting from a microbial environmental source, DON is added as a (sole) carbon source in a minimal medium, therefore, tailoring culture conditions to favor bacteria from the environmental source with particular metabolic activity (DON biotransformation) [18,19]. Via this method, the high diversity of the originally microbial source is profoundly reduced by enhancing the growth of potentially functional strains or suppressing unwanted ones [18,19]. Multiple enrichment cultures and isolates capable of biotransforming DON have been obtained from microbial environments and are reported in the literature (Table 1). Biotransforming of DON occurs mainly through de-epoxidation, oxidation or isomerization. The best-known microbial metabolites of DON are de-epoxy-deoxynivalenol (DOM-1) and 3-epi-DON, which were intensively studied for residual toxicity and proven to be non-toxic [20]. In addition, to evaluate the efficacy of mycotoxin-biotransforming agents, a multi-tiered interdisciplinary approach is required for their in vitro assessment. This assessment involves microbial enrichment and appropriate analytical methods, preferably combined with toxicity assays for the evaluation of residual toxicity [21].

In the current study, different bacterial microbial products were screened for their DON biotransformation potential via an enrichment method. The mycotoxin DON and its potential metabolites were first monitored with HPLC-UV. These metabolites were subsequently in-depth characterized with liquid chromatography high-resolution mass spectrometry (LC-HRMS). This in vitro assessment to evaluate the efficacy of DON-biotransforming agents was combined with the implementation of two toxicity assays. First, the residual toxicity was evaluated by a plant bioassay using the aquatic species *Lemna minor* L. [33], and subsequently by the assessment of the cellular viability of eukaryotic intestinal porcine epithelial cells (IPEC-J2). Implementing toxicity assays in biotransformation experiments is of great importance because the residual toxicity of possible metabolites is often overlooked in screening assays for new promising microbial strategies. At last, the bacterial communities of the subsequent enrichment cultures capable of biotransforming DON were identified using metabarcoding based on the V3–V4 fragment of the 16S rRNA gene.

## 2. Results

### 2.1. Obtaining Enrichment Cultures Capable of Biotransforming DON

Five bacterial products in use or in development to apply in agriculture and/or aquaculture (A, B, C, D, E), were used as bacterial inocula for the enrichment of DON biotransforming bacteria. All products were inoculated in minimal medium with 10 mg/L DON for 6 weeks. Samples were analyzed with ELISA and HPLC-UV. Only products B and E were capable of biotransforming DON, resulting in two enrichment cultures, Enr_B1 and Enr_E1, respectively (supplementary files—Figure S1). HPLC-UV analysis revealed no presence of DON (retention time (r_t_) = 3.2 min) after incubation of 6 weeks, and a potential DON metabolite with a slightly longer retention time than DON (r_t_ = 3.6 min) for both enrichment cultures Enr_B1 and Enr_E1. However, ELISA analysis still showed 28 ± 8% and 23 ± 4% residual DON concentration after incubation of 6 weeks for, respectively, Enr_B1 and Enr_E1 (indicating this metabolite could cause cross reactivity with the used ELISA).

**Figure 1:**
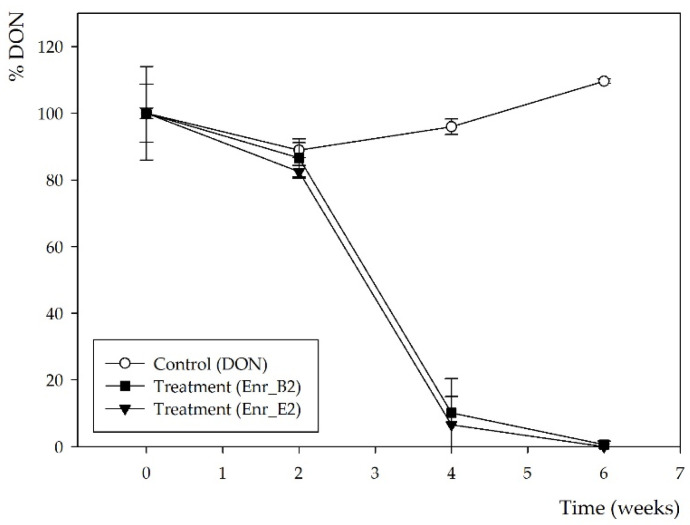
DON biotransformation by microbial enrichment cultures Enr_B2 and Enr_E2: DON concentration measured by HPLC-UV analysis. Values are indicated as mean ± standard error of the mean (*n* = 3).

Subsequently, a second enrichment was performed with both enrichment cultures Enr_B1 and Enr_E1 in minimal medium (MM) and again 10 mg/L DON. More frequent samples over time were now taken, i.e., after 0, 2, 4 and 6 weeks. After 4 weeks, DON was partly biotransformed by both enrichment cultures and the conversion was complete after 6 weeks (Figure 1), resulting in enrichment cultures Enr_B2 and Enr_E2 (supplementary files—Figure S1). Moreover, the same decline was observed in the HPLC-UV chromatograms (supplementary files—Figure S2), where the same potential DON metabolite was observed at r_t_ of 3.6 min, starting from week 4.

**Figure 2:**
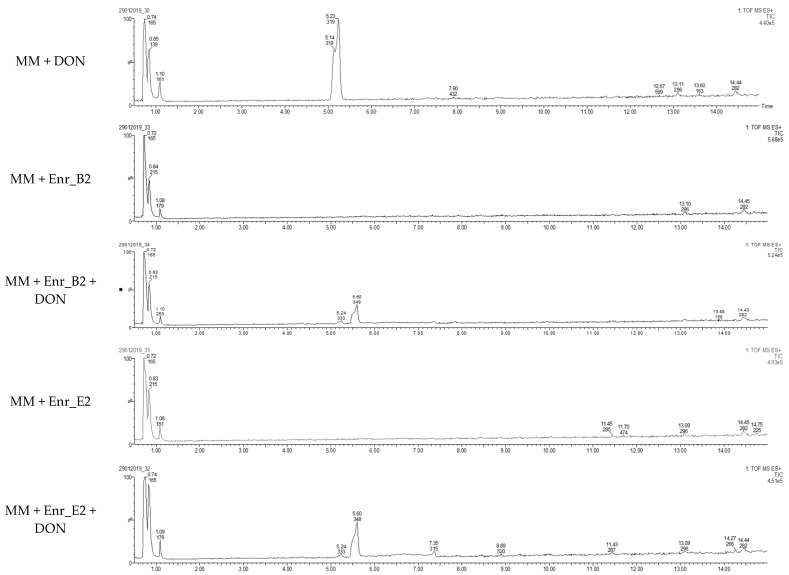
Detection of DON metabolite after DON biotransformation by Enr_B2 and Enr_E2: Extracted ion chromatograms of samples after degradation of DON after 6 weeks by enrichment cultures Enr_B2 and Enr_E2. In both treatments of Enr_B2 and Enr_E2, a metabolite was observed with a retention time longer than DON.

Samples at 6 weeks were also analyzed with LC-HRMS, and for both Enr_B2 and Enr_E2, a metabolite was observed with a retention time (r_t_ = 5.6 min) slightly longer than DON (r_t_ = 5.2 min) (Figure 2). The sodium adduct and the potassium adduct was found with, respectively, mass [M + Na^+^] at *m*/*z* = 349.1263 and [M + K^+^] at *m*/*z* = 365.1263 for both enrichment cultures. The formula was tentatively suggested as C_16_H_22_O_7_ (with mass error = 0.3 ppm and 0.9 ppm, respectively). Product ions at *m*/*z* 309.1308, 279.1202 and 261.1151 were observed in this MS spectrum and the MS^2^ spectra of DON and its metabolite can be found in Figure S3 (supplementary files).

### 2.2. Assessing Residual Toxicity after DON Biotransformation of Enrichment Cultures Enr_B2 and Enr_E2 Using an Aquatic Plant Lemna minor L. Bioassay

The samples were analyzed with the bioassay using *Lemna minor* L. to obtain a first indication of residual toxicity of the potential metabolites (Table 2). Although DON was completely biotransformed after 6 weeks of incubation with both enrichment cultures Enr_B2 and Enr_E2, a toxic effect on the plant growth was observed in presence of the supernatants after biotransformation. Moreover, when only bacteria were added to the medium (MM, negative control), no toxic effect was seen, indicating that the potential metabolite(s) of DON are responsible for the observed high toxicity.

### 2.3. Assessing Residual Toxicity after DON Biotransformation of Enrichment Culture Enr_B2 through Assessment of Eukaryotic Cellular Viability of IPEC-J2

**Figure 3:**
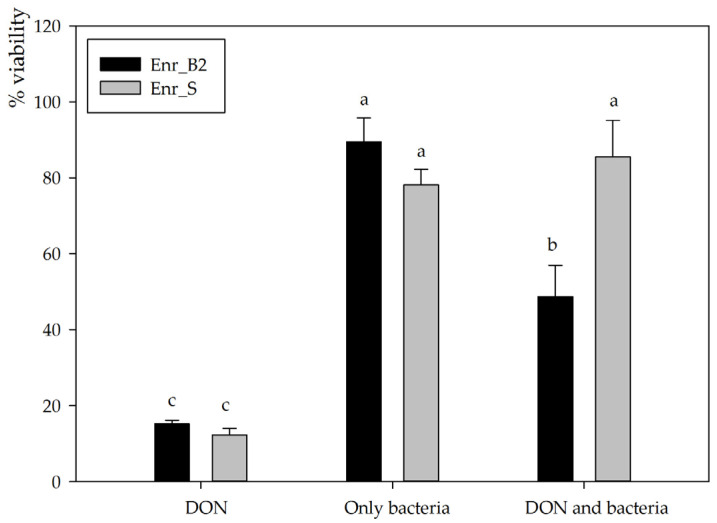
Residual toxicity on IPEC-J2 cells imposed after DON degradation by Enr_B2 and Enr_E2: Cellular viability assessed via neutral red assay of IPEC-J2 cells exposed for 3 days to a final concentration of 5 mg/L DON in MM (in culture medium), to MM treated with only bacteria and to MM in which DON was biotransformed. Biotransformation was performed with two enrichment cultures, Enr_B2 and an in-house additional positive control Enr_S (enriched from soil) known to detoxify DON. All treatments are compared to only MM added to cell line culture medium (=100%). Values are indicated as mean ± standard error of the mean (*n* = 3). ^a,b,c^ Statistically analyzed via a one-way ANOVA test followed by a one-sided post-hoc Tukey HSD test (α: 0.05).

For subsequent experiments, enrichment cultures Enr_B2 and Enr_E2 were used to set up new biotransformation experiments. Enr_B2 was able to biotransform 10, 25 as well as 50 mg/L DON completely in MM after 6 weeks, consistently resulting in the same metabolite (analyzed with HPLC-UV and LC-HRMS). However, Enr_E2 became unable to consistently degrade DON and eventually lost its degradative capability, leading its omission from further experiments. The supernatant after degradation of 25 mg/L DON by enrichment culture B2 was used to investigate the residual toxicity on the IPEC-J2 cell line. Cellular viability was assessed via the neutral red assay exposing proliferative cells to the supernatant diluted to a final concentration of 5 mg/L DON for 3 days. After exposure to DON alone, a significantly decreased viability was observed (15.2 ± 0.9%, *n* = 3), whereas inoculation with bacteria alone did not affect cellular viability (89.5 ± 6.3%, *n* = 3, *p* < 0.001) (Figure 3). The DON-incubated enrichment culture Enr_B2 caused a major decrease in cellular viability (48.7 ± 8.3%, *n* = 3, *p* = 0.001), indicating that the presumed DON metabolite is cytotoxic to eukaryotic IPEC-J2 cells, although to a lesser extent than the mother compound DON. These results for the culture Enr_B2 were compared to an in-house available positive control, i.e., an enrichment culture Enr_S from soil capable of degrading DON into the non-toxic metabolites 3-epi-DON and the epimer of DOM-1 [33]. This complementing biotransformation experiment was also performed at a final concentration of 5 mg/L DON in minimal medium in the assay (Figure 3). The negative control (only DON) revealed the same toxic effect as in the Enr_B2 trail (12.2 ± 1.7%). Neither the bacteria from Enr_S, nor the treatment (DON and Enr_S) had a significant effect on the IPEC-J2 cell viability (78.1 ± 4.1%, *n* = 3, *p* < 0.001; and 85.5 ± 9.6%, *n* = 3, *p* < 0.001, respectively), confirming its detoxification potential.

### 2.4. Metabarcoding of Bacterial Communities during Enrichment of Product B

Subsequently, 16S rRNA metabarcoding of the V3-V4 fragment was performed on the bacterial communities in subsequent enrichments of product B. Ultimately, the start product B was subsequently enriched four times (Enr_B1, Enr_B2, Enr_B3 and Enr_B4), of which the third and fourth enrichment were performed in four biological replicates (Enr_B3_r1–4, and Enr_B4_r1–4; supplementary files—Figure S1). Except for Enr_B4_r3, in all other enrichments, DON was biotransformed and the previously observed metabolite was detected. In total, 359.0 ± 9.0 amplicon sequence variants (ASV’s) were detected in the start product B, which decreased during the four enrichments towards 123.8 ± 9.2 ASVs with DON biotransformation. Accordantly, a significant decrease was observed in microbial diversity (Shannon diversity index), which decreased from 4.35 ± 0.07 in the start product to 2.62 ± 1.17 (*p* = 0.04; Wilcoxon rank-sum test) after the third enrichment and 2.92 ± 0.41 (*p* = 0.04; Wilcoxon rank-sum test) after the fourth enrichment (supplementary files—Figure S4). Notice the high variability in the third enrichment, which could be explained because of a separation in replicates 1 and 2 which show an overall lower diversity compared to replicates 3 and 4.

**Figure 4:**
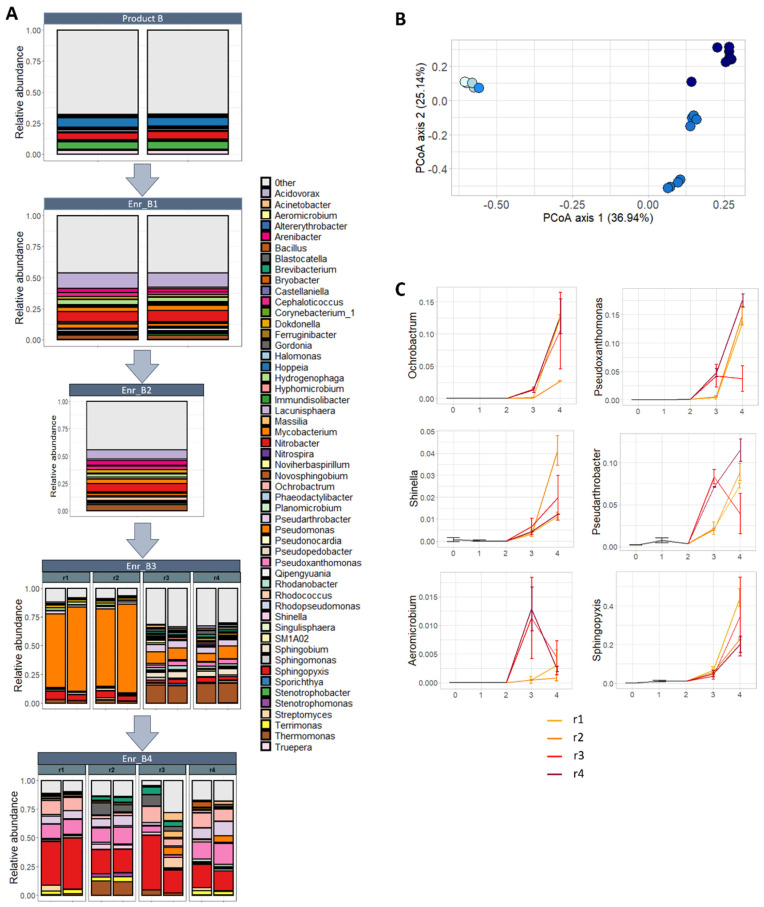
Composition of the bacterial community in subsequent enrichments of product B: (**A**) Relative abundance of genera (accounting for at least 1% in at least 1 sample) per enrichment, starting from product B, first (Enr_B1), second (Enr_B2), third (Enr_B3) and fourth (Enr_B4) enrichment. (**B**) Principle coordinate analysis plot illustrating the effect of the enrichment on bacterial community. The further the enrichment, the darker the dots are colored (start product: light blue → 4th enrichment: dark blue). A shift in the composition of the bacterial community can be noted between the start product and the third and fourth enrichment. (**C**) The abundance of the genera with a statistical significant increase (GLM-NB; *p* < 0.05) during the enrichment: *Ochrobactrum*, *Pseudoxanthomonas*, *Shinella*, *Pseudoarthrobacter*, *Aeromicrobium* and *Sphingopyxis*. The start product, first and second enrichment are illustrated as one black line, corresponding to one biological replicate; the third and fourth enrichment are illustrated as four colored lines, corresponding to four biological replicates (r1–4), of which biological replicate r3 was not able to degrade DON in the fourth enrichment.

Thereafter, the bacterial community composition on a genus level was determined per enrichment, starting from the start product B towards the four enrichments (Figure 4A). For the start product, 290 genera were detected in total; however, for visualization, only 51 are shown, which represent all genera with an abundance of at least 1% in either of the enrichments or the start product (supplementary files—Table S1). After the first, third and fourth enrichment, a reduction was observed to, respectively, 206, 151 and 167 genera in total. In replicates r1 and r2 of the third enrichment, an extremely high abundance of *Pseudomonas* was observed, whereas the genus *Thermomonas* also was present in high incidence in replicates r3 and r4, followed by *Pseudomonas*. After the fourth enrichment, a high abundance of *Sphingopyxis* was observed in all four biological replicates, followed by a high percentage of *Pseudoxanthomonas* in the three biological replicates where DON was degraded.

Lastly, differences between samples in community composition were studied. An extreme significant effect of the enrichments on the bacterial community was observed (*p* < 0.001; PERMANOVA), illustrated in the principal coordinate analysis (PCoA) (Figure 4B). Whereas no change in the bacterial community was noted between the start product, the first and second enrichment, a major shift was seen between the start product/1st/2nd enrichment and the 3rd/4th enrichment, accounting for almost 37% of the total variance. In addition, there was a differentiation between the parallel biological lineages of the third enrichment (Enr_B3r1–2, and r3–4) and the fourth enrichment (second axis, 25.14%). In the fourth enrichment, the separation between the parallel enrichment lineages (Enr_B4r1–4) was not really pronounced, however.

No bacterial genera were shifting between the start to the first enrichment. The difference in community composition between the first enrichment and third enrichment was explained by the decrease in relative abundance of 44 genera and the increase of five genera (10.4× *Ochrobactrum*, 10.1× *Aeromicrobium*, 7.6× *Pseudoxanthomonas*, 4.4× *Shinella* and 2.4× *Pseudarthrobacter*). Between the third and fourth enrichment, changes were less pronounced: five genera decreased and six increased in abundance (supplementary files—Table S2), including also the genera *Ochrobactrum*, *Pseudoxanthomonas*, *Shinella* and *Pseudarthrobacter* (Figure 4C). In addition, 42 bacterial genera decreased and eight increased in abundance between the first and fourth enrichment. The genus *Sphingopyxis* had an abundance of 40% after the fourth enrichment, whereas *Pseudoxanthomonas* and *Ochrobactrum* had over 10%. Interestingly, the biological replicate r3 of the fourth enrichment reacted differently for the genera *Pseudoxanthomonas* and *Pseudarthrobacter*, compared to r1, r2 and r4, showing there might be a difference in those treatments.

## 3. Discussion

In this study, five bacterial products were enriched on minimal medium with DON and screened for DON biotransformation and detoxification, resulting in two enrichment cultures, Enr_B1 and Enr_E1, capable of complete biotransformation of DON after six weeks. In the second enrichment, resulting in the DON biotransformation enrichment cultures Enr_B2 and Enr_E2, a potential new bacterial metabolite of DON was characterized with LC-HRMS, suggesting C_16_H_22_O_7_ as its elemental formula. Residual toxicity of these supernatants after biotransformation was investigated with a bioassay using the aquatic plant *Lemna minor* L. and further determined by a cytotoxicity assay assessing the cellular viability of the eukaryotic IPEC-J2 cell line. In both assays, a phyto- and cytotoxic effect was observed, probably caused by the bacterial formation of the DON metabolite. Metabarcoding of the 16S rRNA gene of the bacterial communities in subsequent enrichments of product B revealed a major shift towards mainly the genera *Sphingopyxis*, *Pseudoxanthomonas*, *Ochrobactrum* and *Pseudarthrobacter*.

Our above-proposed novel bacterial metabolite has not been reported, as far as we know, in the literature. The main described microbial conversions of DON are de-epoxidation, oxidation and epimerization (Table 1), leading to the metabolites DOM-1, 3-keto-DON and 3-epi-DON. The formation of the latter two metabolites were frequently accompanied with the detection of yet-unidentified products. Moreover, two less-common conversions have been reported once in the literature, resulting in the metabolite 16-hydroxy-deoxynivalenol (16H-DON) [51] and a biotransformation product of DON with a molecular weight of 18.1 Da (H_2_O) higher than DON [50]. In some other reports, either no DON metabolite was found, or no adequate detection technique was used to characterize possible metabolites [52,53]. However, no similarity has been found in the literature with the found microbial DON derivative in this current study. All known biotransformation products are summarized in supplementary files (Table S3) with their corresponding elemental formula, structure, precursor and product ions, for comparison with the presumed novel metabolite found in the current study. Importantly, if we compare our metabolite with known, either chemically or thermally formed, metabolites of DON, a similarity can be found only with a thermal degradation product of DON, 9-hydroxymethyl DON lactone, which has the same elemental formula as the found metabolite in the current study (C_16_H_22_O_7_) [54]. However, only one product ion (*m*/*z* 309) is similar and indicates the loss of a water molecule, while the other two product ions (*m*/*z* 279 and 261) of the found metabolite in this current study corresponded with product ions originating from the parental molecule DON. This indicates a resemblance with the core structure of DON. Contrarily, the thermal product 9-hydroxymethyl DON lactone is a complex derivate of DON where the well-known C12-13 epoxide ring was disrupted (supplementary files—Table S3). A functional group that matches the surplus molecular weight of our metabolite compared to DON is an aldehyde group which, when added to the native DON molecule at the C10 position, results in the proposed structure in Table S3. We suspect that the electron dense double bond at C9-10 can attack the partially positively charged carbon atom of an aldehyde, thereby being reduced. However, this hypothesis needs to be confirmed and validated. This microbial metabolite is, to our knowledge, for the first time reported in the literature.

Regarding the unexpected residual toxicity of our enrichment cultures, all known microbial conversions in the literature resulted in a decrease in toxicity compared to the native DON molecule. Some of them were even tested several times in different assays and were proven to be non-toxic, such as DOM-1 and 3-epi-DON (supplementary files—Table S4). Others were slightly less investigated, such as 3-keto-DON, which only resulted in a small decrease in toxicity, depending on the assay. Little information is currently available about the residual toxicity of the microbial metabolites, the epimer of DOM-1, 16-HDON and DON derivate + MW(H_2_O). In addition, no toxicity studies have been performed yet with the thermal degradation product of DON, 9-hydroxymethyl DON lactone. Only for the epimer of DOM-1 and 16H-DON, an indication of a reduced phytotoxicity in two different bioassays has been reported (Table S4). In present study, the supernatant showed both a phyto- and cytotoxic effect, presumably caused by the metabolite DON + MW(CH_2_O). Pierron et al. [20] investigated the molecular basis for the reduced toxicity of DOM-1 and 3-epi-DON, more specifically, the ability of DON, DOM-1 and 3-epi-DON to bind to the A site of the ribosome peptidyl transferase during the elongation step of protein translation. According to an in silico analysis, DON forms three hydrogen bonds with the A site of the ribosome 60S subunit. The first one is between the oxygen of the DON epoxy group on C12 and one hydrogen of the ribose from uracil U2873; the second one is between the oxygen of the C15 group CH_2_OH and one hydrogen of guanine G2403; and the last one is between the hydrogen of the C3 group and one oxygen of uracil U2869. This in silico analysis further revealed that both DOM-1 and 3-epi-DON were able to fit into the pocket of the peptidyl transferase active center. However, because of the absence of the epoxy group or the isomeric change, these two metabolites were only able to form two weak non-covalent hydrogen bonds with the A site of the peptidyl transferase active center. Taking this into account, if an aldehyde group was added to the new metabolite found in this study on the C10, it could be that the molecule still fits into the pocket of the peptidyl transferase center, forming three hydrogen bonds including the oxygen of the epoxide group and the hydrogen of the C3 group, thus causing its toxicity. Follow-up research is needed to purify and identify the molecule to perform multiple toxicological characterization studies and to investigate the molecular basis for the toxicity. To the best of our knowledge, this is the first study reporting a microbial conversion of DON to a toxic metabolite. In contrast, several microbial and in vivo metabolites of the mycotoxin zearalenone (ZEN) are known to still induce a high or slightly lower estrogenic activity (alpha-zearalenol (α-ZEL) and beta-zearalenol (β-ZEL), respectively) [55,56,57,58,59,60]. Therefore, a bio- or cytotoxicity assay is recommended to include in biotransformation assays to obtain a first indication of residual toxicity of the microbial metabolites [61].

Out of the five start products, which have no apparent connection to DON exposure, products B and E, were able to biotransform the mycotoxin completely, starting from the first enrichment. During the subsequent enrichments of product B, a shift in bacterial community was observed towards mainly the genera *Sphingopyxis*, *Pseudoxanthomonas*, *Ochrobactrum* and *Pseudarthrobacter*, which are known for their biodegradation capabilities of complex molecules, such as (polycylic) aromatic hydrocarbons [62,63,64,65,66,67,68], neonicotinoid insecticides [69], cyanobacterial toxins [66,70,71], phthalate esters [72], polyhydroxyalkanoates [73], nitrophenols [74], 2,4,6-trinitrotoluene [75], etc. The classes to which these genera belong, the α-, γ-proteobacteria and Actinobacteria, are also linked to the bioremediation and biodegradation of other mycotoxins such as aflatoxins, fumonisins, zearalenone and ochratoxins [76,77,78,79]. Furthermore, some similarities have been found of detected genera in our enrichment cultures, compared to DON-biotransforming organisms, already reported in the literature (Table 1). First of all, *Sphingopyxis* was present during all subsequent enrichments of B, especially high in abundance in the fourth enrichment. The DON degrading consortium IFSN-C1, biotransforming DON to 3-keto-DON and 3-epi-DON was also predominantly composed of the genera *Sphingopyxis*, *Sphingomonas* and *Achromobacter* [35]. In our enrichment culture Enr_B, *Pseudomonas* is present in high abundance in the third enrichment, whereas Zhai et al. [36] obtained an enrichment culture degrading DON into 3-epi-DON with a high abundance of *Pseudomonas* and *Marmoricola*, and a microbial culture was found by He et al. [25] capable of biotransforming DON into DOM-1, also with a high abundance of *Pseudomonas*. Ahad et al. [24] was able to achieve a highly enriched microbial consortium DX100 dominated by *Stenotrophomonas*, capable of transforming eleven trichothecenes to their less toxic de-epoxy forms, whereas *Stenotrophomonas* was present in our culture Enr_B in the fourth enrichment. In the microbial culture of Islam et al. [23], capable of biotransforming DON into DOM-1, *Stenotrophomonas* was also present, next to a high abundance of *Serratia*. Lastly, a microbial culture from soil capable of degrading DON into 3-keto-DON contained mostly members of the genera *Acinetobacter*, *Leadbetterella* and *Gemmata* [34], whereas *Acinetobacter* was also found in all four enrichments of product B. In addition, *Sphingomonas* was present in the third enrichment, which is known to degrade DON into 3-keto-DON, 3-epi-DON or 16-HDON [48,51]. *Bacillus*, also present in the fourth enrichment, is known to degrade DON into DOM-1 or not (yet) defined metabolites [28,53]. Despite these similarities between previously known genera that convert DON, and the composition of our enrichment cultures, it is peculiar that our enrichment cultures converted DON into a toxic metabolite, which has never been reported before.

## 4. Conclusions

To conclude, the enrichment method seemed to be highly effective to obtain DON biotransforming organisms from diverse microbial products. However, this research resulted in the discovery of two bacterial enrichment cultures able to biotransform DON into a previously undescribed metabolite, toxic to plant and porcine cells. LC-HRMS analysis tentatively characterized this metabolite with a molecular weight of 30.0 Da (CH_2_O) higher than DON. With the implementation of two toxicity assays in the in vitro assessment of the efficacy of mycotoxin-biotransforming agents, the residual toxicity was rapidly detected and seemed to be a valuable tool to implement in biotransforming assays. At last, metabarcoding of the bacterial communities on the V3-V4 fragment of the 16S rRNA gene revealed a shift in subsequent enrichments of product B towards mainly the genera *Sphingopyxis*, *Pseudoxanthomonas*, *Ochrobactrum* and *Pseudarthrobacter*, indicating that these genera could be responsible for this particular DON biotransformation.

## 5. Materials and Methods

### 5.1. Bacterial Sources

Five bacterial products (A, B, C, D, E), were used as bacterial inocula for the enrichment of DON-biotransforming bacteria. Only two products are commercialized, i.e., product B corresponding to ABIL^®^ (Avecom, Ghent, Belgium) and product C to ProMic^®^ (Avecom, Ghent, Belgium). The other products are still in development to apply in agriculture, of which the exact composition is not known. Product A promotes plant growth and can be added to soil. Product B consists of mainly ammonia and nitrite oxidizing bacteria, which is originally applied as enhancer of water quality in aquaculture systems. Product C and D are based on aerobic heterotrophic bacteria and can be used as protein extracts for animal feed. Product E was enriched in straw with a mix of mainly heterotrophs and some autotrophs to apply as a nitrogen source to feed.

Solid product A was diluted in sterile physiological water (8.5 g/L NaCl in distilled water) to 82.4% (*w*/*v*) (41.2 g in 50 mL) and homogenized in a stomacher for 2 min where the liquid phase was separated from the solid form. Product B and C are liquid samples, and no preparation was therefore needed. Product D was diluted with physiological water to 10% (*w*/*v*) and vortexed thoroughly. Product E was diluted in physiological water to 32.8% (*w*/*v*) and treated in a stomacher for 5 min. All final bacterial liquid phases were centrifuged, and the pellet was washed with physiological water four times to remove impurities.

### 5.2. Standards

The individual mycotoxin solid calibration standards (1 mg) of DON, 3-acetyl-deoxynivalenol (3-ADON), 15-acetyl-deoxynivalenol (15-ADON) and DOM-1 were purchased from Sigma-Aldrich (Bornem, Belgium). Deoxynivalenol-3-glucoside (DON-3G) (50.2 ng/µL, in acetonitrile) was purchased from Biopure Referenzsubstanzen (Tulln, Austria). All mycotoxin solid standards for HPLC-UV and LC-HRMS analysis were dissolved in methanol (1 mg/mL), and were storable for a minimum of 1 year at −18 °C [80]. DON-3G was kept at 4 °C. Working solutions of DON standard for the enrichment protocol and the bioassay were prepared as 100 and 10 mg/L in MilliQ water and stored at −18 °C.

### 5.3. Enrichment of DON-Biotransforming Microorganisms

To obtain DON-biotransforming organisms, the enrichment method was used where DON was added as sole carbon source into a minimal medium (MM), prepared according to Stanier et al. [81]. The medium consisted of 1.4 g/L Na_2_HPO_4_, 1.4 g/L KH_2_PO_4_, 0.3 g/L (NH_4_)_2_SO_4_, 98.5 mg/L MgSO_4_, 5.9 mg/L CaCl_2_·H_2_O, 3.2 mg/L Na_2_EDTA, 2.8 mg/L FeSO_4_·7H_2_O, 1.2 mg/L ZnSO_4_·7H_2_O, 1.7 mg/L MnSO_4_·H_2_O, 0.4 mg/L CuSO_4_·5H_2_O, 0.2 mg/L CoCl_2_·6H_2_O and 0.1 mg/L (NH_4_)_6_Mo_24_·4H_2_O. The prepared bacterial inoculants originating from products A, B, C, D and E were added in triplicate at 1% in MM with 10 mg/L DON in a total volume of 10 mL. These mixtures were incubated for 6 weeks at 30 °C (100 rpm) to enrich potential DON-biotransforming organisms. A negative control was included in triplicate (MM + 10 mg/L DON). Samples were taken at time points of 0 and 6 weeks (V = 1.5 mL) and stored at −18 °C. Enrichment cultures were stored at −80 °C in 20% (*v*/*v*) glycerol.

Enrichment cultures capable of biotransforming DON were enriched further under similar conditions. First, they were grown as preculture in MM and 10 mg/L DON, whereas 1% of bacterial suspension of the preculture was added into 10, 25 or 50 mg/L DON in MM in a total volume of 12 mL and incubated for 6 weeks at 30 °C (100 rpm). Two controls were included (1. MM + corresponding concentrations of DON and 2. MM + bacterial suspension). These experiments were performed in triplicate or four replicates. Samples were taken at time points of 0, 2, 4 and 6 weeks (V = 2 mL) if necessary, and stored at −18 °C to analyze afterwards with HPLC-UV, LC-HRMS, the bioassay and/or cytotoxicity assay. After biotransformation of DON, bacterial cultures were archived at −80 °C in 20% (*v*/*v*) glycerol.

### 5.4. Analysis of DON Concentration

First, samples were analyzed with an AgraQuant^®^ Deoxynivalenol Assay 0.25/5.0 ELISA kit (Romer Labs, Tulln, Austria) to determine the concentration of DON. Subsequently, HPLC-UV was used to confirm the results, to exclude any cross-reactivity from possible metabolites with the used ELISA and to discover tentatively metabolites.

As preparation for the HPLC-UV analysis, samples were centrifuged for 5 min at 2000× *g*. The supernatant was filtered with a VWR^®^ Sterile Syringe Filter (0.22 µm, VWR, Radnor, PA, USA), diluted with methanol to a final concentration of 17.5% and vortexed for 20 s. Subsequently, samples (V = 10 µL) were injected into a reversed phase HPLC system (1100 Series: Agilent, Santa Clara, CA, USA) with UV detection. Chromatographic separation was performed using a Nova-Pak C18 column (4 µm, 3.9 × 150 mm) and a guard column of the same material (Nova-Pak^®^ C18, 4 µm, 3.9 × 20 mm) (Waters, Wilmslow, UK). The column was kept at 40 °C. The mobile phase contained methanol and water (17.5:82.5, *v*/*v*) at a flow rate of 1.0 mL/min with an isocratic elution for 10 min. A wavelength of 220 nm was used for UV detection. Data were processed with Agilent ChemStation software. The analytical method was validated according to Commission Decision 2002/657/EC [82] and all validated parameters met the criteria mentioned. Validation data for DON in MM are shown in the supplementary files (Table S5). Limit of detection (LOD) and limit of quantification (LOQ) of DON were, respectively, 0.21 mg/L and 0.35 mg/L DON.

### 5.5. LC-HRMS Analysis

All the LC-HRMS analyses were conducted on Synapt G2-Si HDMS instrument, a high-definition hybrid quadrupole time-of-flight (Q-TOF) mass spectrometer equipped with an electrospray ionization (ESI^+/−^) source from the Waters company (Waters, Wilmslow, UK).

Sample preparation was performed just before the LC-HRMS analysis. In brief, the samples (each 200 µL) were evaporated until complete dryness under nitrogen gas at 60 °C using the TurboVap^®^ LV device (Biotage, Dusseldorf, Germany). Afterwards, the samples were re-dissolved in 200 µL of injection solvent (methanol/water (30.7/69.3, *v*/*v*) containing 0.035% HCOOH and 3.5 mM HCOONH_4_, vortexed for 2 min and transferred into an Ultrafree-MC centrifugal tube (0.22 µm, Millipore, Bedford, MA, USA) for centrifugation for 5 min at 10,000× *g*. After centrifugation, samples were transferred into injection vials for the untargeted analysis.

The Synapt G2-Si HDMS instrument was controlled using the commercial software Masslynx 4.1 (Waters, Wilmslow, UK) which was also used for data processing. Chromatographic separation was achieved on an ACQUITY UPLC I-class FTN system (Waters, Wilmslow, UK) using ACQUITY UPLC HSS T3 column. The mobile phase consisted of H_2_O:MeOH (99:1, *v*/*v*) containing 0.05% HCOOH and 5 mM HCOONH_4_ (solvent A) and MeOH (solvent B). A gradient elution program was adjusted as follows: 0–0.5 min: 5% B, 0.5–20 min: 5–95% B, 20–21 min: 95% B, 21–24 min: 95–5% B, 24–28 min: 5% B. The flow rate was 0.3 mL/min. The injection volume of the sample was three µL and the flow rate of the mobile phase was 0.3 mL/min. Temperature of the column and the autosampler were set at 40 °C and 7 °C, respectively.

Data acquisition was on positive ion polarity (ESI^+^) in resolution mode (>20,000 full width half maximum (FWHM)) [33]. The parameters for MS were applied as follows: capillary voltage 2.8 kV; sample cone voltage 40 V; source temperature 150 °C; desolvation gas flow 800 L/h at a temperature of 550 °C and cone gas flow 50 L/h. Sodium formate clusters were used for instrument calibration before running the samples. Nitrogen and argon were used as, respectively, desolvation gas and collision gas at a pressure of 9.28 × 10^−3^ mbar. Leucine-enkephalin solution (200 pg/µL) was continuously infused during the analysis into the MS via the lock spray interface at a flow rate of 20 μL/min, generating the reference ion ([M + H]^+^ at *m*/*z* = 556.2771) for mass correction. Mass spectra were collected in continuum mode from *m*/*z* 50 to 1200 with a scan time of 0.2 s, an inter-scan delay of 0.01 s and a lock spray frequency of 20 s with a mass window of ± 0.5 Da. A data-dependent acquisition (DDA) mode was implemented to obtain the simultaneous acquisition of exact mass data for the precursor and fragment ions. From a single MS survey scan, only the top five ions were selected for the next MS/MS fragmentation. The scan time for MS/MS was 0.1 s. The collision energy in the trap cell was ramped from 10/15 V (low mass, start/end) up to 60/150 V (high mass, start/end).

### 5.6. Bioassay Using Lemna minor L.

The protocol was based on Vanhoutte et al. [33]. A growth medium (GM) for the plant *Lemna minor* L. was prepared according to Megateli et al. [83]. A sterile 24-well plate was used for the growth of *Lemna minor* L. in GM, in a total volume of 2 mL per well. Starting from six fronds, the plants were incubated in four replicates for 7 days in a growth chamber (16 h light exposure, 22 °C). For each analysis, controls were included (0 and 1 mg/L DON). Samples were filter sterilized using a sterile filter (0.45 µm) and syringe. Samples (containing e.g., 10 mg/L DON) were 10 times diluted in GM to a final concentration of 1 mg/L in the bioassay. After 7 days of incubation, the plants were analyzed based on frond growth. The number of fronds were counted with a microscope (Stereo IX). Photos were made of each well with the microscope and frond area was calculated with the program APS Assess. The growth was expressed as

%growth = growth_sample_/growth_control_ × 100%. (1)

Data were statistically analyzed via a one-way ANOVA test followed by a one-sided post-hoc Dunnett’s test (α: 0.05).

### 5.7. Cytotoxicity Assay Using IPEC-J2 Cell Line: Neutral Red Assay

#### 5.7.1. Cell Line and Culture Conditions

The IPEC-J2 cell line is a continuous intestinal cell line derived from the jejunal epithelium isolated from a neonatal piglet [84]. The IPEC-J2 cells are derived from the small intestine and are neither transformed nor tumorigenic in nature [85]. These cells were maintained in 1:1 Dulbecco’s Modified Eagle Medium (DMEM)/Ham’s F-12 Medium (Thermo Fisher Scientific, Waltham, MA, USA), supplemented with 5% (*v*/*v*) Fetal Calf Serum (FCS) (Hyclone, Cramlington, England, UK), 1% (*v*/*v*) Insulin-Transferrin-Selenium supplement (Gibco, Life Technologies, Paisley, Scotland, UK), 1% (*v*/*v*) penicillin-streptomycin (Gibco) and 1% (*v*/*v*) kanamycin (Gibco), further called culture medium [86]. Cells were incubated at 37 °C in ambient atmosphere with 5% CO_2_.

Cells were maintained in plastic tissue culture flasks (75 cm^2^, Nunc, Denmark) and passaged twice weekly. Cells were trypsinized using 0.25% Trypsine with 0.02% EDTA. Detached cells were collected, counted with a Bürker counting chamber (Karl Hecht, Sondheim vor der Rhön, Germany) and added at 0.88 × 10^6^ cells into a new flask for further growth. Bacterial and mycoplasma contamination were tested several times during cell culture using HEK-Blue^TM^-2 cells in the PlasmoTest^TM^ (Invivogen, Toulouse, France), obtaining a negative result each time.

#### 5.7.2. Cell Culture Assay

For the cytotoxic assay, the IPEC-J2 cells were seeded at 1 × 10^5^ cells/well on 24 well plates in 1 mL of culture medium and allowed to grow for 3 days (proliferative cells) [86]. Each well was washed with 1 mL of sterile Hanks’ Balanced Salt solution (HBSS) (Gibco) in order to remove dead cells. Monolayers were exposed for 3 days to DON or MM (controls) and to the filtered (0.22 µm) supernatant of the biotransformation experiments with enrichment cultures in MM and 10 mg/L DON. The controls and supernatant were 5 times diluted into culture medium. All treatments were conducted in triplicate and incubated in a humidified environment (37 °C, 5% CO_2_).

#### 5.7.3. Neutral Red Assay (NR)

Cell viability was measured using the neutral red assay. The culture medium was removed, and the monolayer was rinsed with 1 mL DMEM without phenol red (Gibco). In each well, 200 µL was added with 33 mg/L Neutral Red solution (Sigma-Aldrich, St. Louis, MO, USA) (diluted in DMEM without phenol red). After incubation of 2 h in the dark at 37 °C, cells were washed two times with HBSS and 200 µL extraction solution (ethanol/water/acetic acid, 50/49/1, *v*/*v*/*v*) was added to each well. The plate was shaken for 10 min at 200 rpm at room temperature. Subsequently, the absorbance was measured at 540 nm using a microplate ELISA reader (Multiscan MS, Thermo Labsystems, Helsinki, Finland). Viability was calculated according to following formula [87]:

%viability = (A_sample_ − A_no cells_)/(A_control_ − A_no cells_) × 100%, (2)

where A = absorbance, A_sample_ = derived from treatment in degradation experiment, A_control_ = derived from untreated degradation experiment (only MM) and A_no cells_ = derived from blank inserts without cells. Data were statistically analyzed via a one-way ANOVA test followed by a one-sided post-hoc Tukey HSD test (α: 0.05).

### 5.8. Microbial Analysis

Metabarcoding was performed on the V3-V4 fragment of the 16S rRNA gene of the bacterial communities of the subsequent enrichments of product B. In total, the start product B was enriched four times during time (Enr_B1, Enr_B2, Enr_B3 and Enr_B4), of which the third and fourth enrichment were performed in four biological replicates (Enr_B3_r1, Enr_B3_r2, Enr_B3_r3, Enr_B3_r4, Enr_B4_r1, Enr_B4_r2, Enr_B4_r3 and Enr_B4_r4) (supplementary files—Figure S1). With the exception of Enr_B2, two technical replicates were taken from each enrichment, resulting in 2 samples for the start product and Enr_B1, 1 sample for Enr_B2 and eight samples for Enr_B3 and Enr_B4. All bacterial samples were stored at −80 °C in 20% (*v*/*v*) glycerol and subsequently DNA was extracted using the DNeasy^®^ PowerSoil^®^ Kit (Qiagen, Hilden, Germany) following the manufacturer’s guidelines. Metabarcoding of the bacterial community was performed on the V3–V4 fragment of the 16S rRNA gene (Illumina, San Diego, CA, USA). Library preparation, quality control, and pooling was performed as described by De Tender et al. [88]. Resulting libraries were sequenced using Illumina MiSeq v3 technology (2 × 300 bp), by Admera Health (South Plainfield, NJ, USA), spiked with 30% PhiX DNA. Demultiplexing of the raw sequences reads was performed by the sequencing provider. Trimming, filtering, merging of the reads, dereplication, sorting, ASV calling and chimera removal were performed as described by De Tender et al. [89]. After dereplication (removal of duplicate reads), reads were merged and an ASV table was built making use of the DADA2 pipeline [90]. To assign taxonomy through the decipher library (v2.12.0) to the resulting ASVs, the SILVA database v132 [91] for the V3-V4 16S rRNA gene sequences was used as a reference. The resulting count table was used for statistical analysis.

Before starting analysis, the ASV table was filtered by which only reads were kept with a count of 2 in at least 2 samples. To study the alpha-diversity of the Shannon-diversity index was calculated with the diversity function of the vegan package in R (version 2.5–6) [92]. For visualization, the most common genera were used (present > 1% in the sample). Absolute ASV counts were transformed to relative abundances and clustered at genus level. Next, differences in community composition were studied. A dissimilarity matrix was made from the ASV table based on the Bray–Curtis dissimilarity index. Using the betadisper function, the homogeneity of the variances was checked on this dissimilarity matrix. As this was fulfilled and thus the variances between enrichments were the same, we could assess the effect of enrichment by doing a PERMANOVA analysis on the dissimilarity matrix. Community differences were visualized by principal coordinate analysis (PCoA) on the dissimilarity matrix. At last, the effect of enrichment of product B was tested using the edgeR package (version 3.28.0) [93] as described in detail in De Tender et al. [94]. According to Pot et al. [95], normalization based on the trimmed mean of M-values (TMM) was applied to correct for differences in library size of the count table. A design matrix was defined based on the experimental design. The dispersion parameter was calculated. Following, a negative binomial model was fitted for every ASV and then combined. Likelihood-ratio tests were conducted on the contrast of the model parameters to assess differential abundances. *p*-values less than 0.05 and log2 fold changes smaller than −2 and larger than 0.5 were considered significant. All visualizations and analyses were performed in R (version 4.3) with the ggplot2 package.

## Figures and Tables

**Table 1:** Microbial biotransformation of DON.

Metabolite(s) of DON	Microbial Culture or Isolate Reported to Biotransform DON	References
DOM-1	Microbial culture C133	[22]
	Microbial culture (mainly *Serratia*)	[23]
	Microbial culture DX100 (mainly *Stenotrophonomas*)	[24]
	Microbial culture PGC-3 (mainly *Desulfitobacterium*)	[25]
	*Eubacterium* BBSH 797	[26,27]
	*Bacillus* LS-100 *Anaerofilum* LS-83 *Anaerofilum* LS-72 *Clostridiales* LS-61 *Coriobacterium* LS-94 *Collinsella* LS-121 *Collinsella* LS-129	[28]
	*Eggerthella* sp. DII-9	[29]
	*Slackia* sp. D-G6	[30]
	*Citrobacter freundii* ADS47	[31]
3-keto-DON and/or 3-epi-DON	Microbial culture D107	[32]
	Microbial culture	[33]
	Microbial culture 1 (mainly *Acinetobacter*) Microbial culture 2 (mainly *Leadbetterella, Gemmata*)	[34]
	Microbial culture IFSN-C1 (mainly *Achromobacter, Sphingopyxis, Sphingomonas*)	[35]
	Mixture *Pseudomonas* sp. Y1 and *Lysobacter* sp. S1	[36]
	*Pelagibacterium halotolerans* ANSP101	[37]
	*Agrobacterium-Rhizobiu*m E3-39 (~*Devosia*)	[38,39]
	*Devosia mutans* 17-2-E-8	[40,41,42,43,44]
	*Devosia* sp. strain D6-9	[45]
	*Devosia insulae* A16	[46]
	*Paradevosia shaoguanensis* DDB001	[47]
	*Devosia* sp.	[38]
	*Sphingomonas* strain S3-4	[48]
	*Nocardioides* sp.	[38]
	*Nocardioides WSN05-2*	[49]
DON + MW(H_2_O)	*Aspergillus Tubingensis*	[50]
16-hydroxy-DON	*Sphingomonas* KSM1	[51]
/	*Marmoricola* MIM116	[52]
	*Bacillus licheniformis* YB9	[53]

**Table 2:** Residual toxicity on *Lemna minor* L. imposed after DON degradation by Enr_B2 and Enr_E2: Analysis of residual toxicity with the screening bioassay using the aquatic plant *Lemna minor* L. expressed as relative growth (%).

Treatment	Relative Growth of *Lemna minor* L. (%) in Relation to MM			
	Time (Weeks) 0	2	4	6
MM + DON	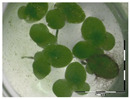 41 ± 4 ^b^	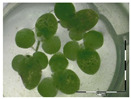 39 ± 1 ^b^	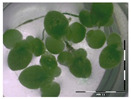 40 ± 9 ^b^	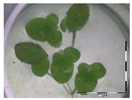 30 ± 1 ^b^
MM + Enr_B2	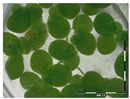 97 ± 4 ^a^	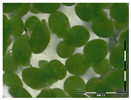 103 ± 4 ^a^	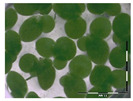 106 ± 1 ^a^	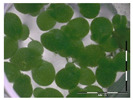 97 ± 7 ^a^
MM + Enr_B2 + DON	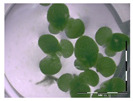 44 ± 8 ^b^	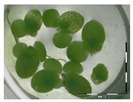 41 ± 3 ^b^	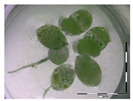 26 ± 3 ^b^	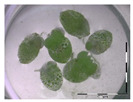 30 ± 5 ^b^
MM + Enr_E2	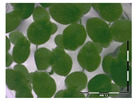 97 ± 4 ^a^	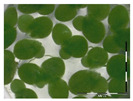 106 ± 6 ^a^	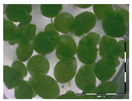 99 ± 4 ^a^	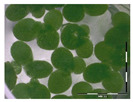 97 ± 14 ^a^
MM + Enr_E2 + DON	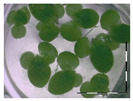 52 ± 6 ^b^	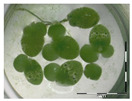 42 ± 4 ^b^	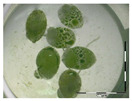 21 ± 3 ^b^	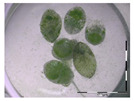 15 ± 0 ^b^

Legend: black-white bar = 5 mm. ^a,b^ Statistically analyzed via a one-way ANOVA test followed by a one-sided post-hoc Dunnett’s test (α: 0.05), each at one time point.

## Supplementary Materials

The following are available online at https://www.mdpi.com/article/10.3390/toxins13080552/s1, Figure S1. Scheme of experimental setup of enrichment of microbial products A, B, C, D and E; Figure S2. Chromatograms of HPLC-UV analysis at week 0, 2, 4 and 6 of samples treated with enrichment cultures Enr_B2 and Enr_E2; Figure S3. MS/MS spectra of DON (red) and the new metabolite with elemental formula C_16_H_22_O_7_ (green); Figure S4. Evaluating the α-diversity: differences between the start product B/1st/2nd enrichment, the 3rd and 4th enrichment in richness versus diversity; Table S1. Relative abundance of genera per enrichment, starting from product (B), first (Enr_B1), second (Enr_B2), third (Enr_B3) and fourth (Enr_B4) enrichment (only visualization of genera with abundance above 1%); Table S2. Difference in community composition between 1st and 3rd enrichment, 3rd and 4th enrichment, and 1st and 4th enrichment (expressed as increase in relative abundance); Table S3. Metabolites of DON with corresponding elemental formula, structure, precursor and product ions in positive mode in LC-MS/MS analysis; Table S4. Phyto- or cytotoxic assays performed on DON metabolites; Table S5. Validation parameters for DON in minimal medium (HPLC-UV analysis).
