# Supplementary material for: Bacterial Enrichment Cultures Biotransform the Mycotoxin Deoxynivalenol into a Novel Metabolite Toxic to Plant and Porcine Cells
Source: Toxins (Basel). 2021 Aug 9;13(8):552. doi: 10.3390/toxins13080552 (PMC8402469; doi:10.3390/toxins13080552)
Supplement: Supplementary file 1 [file toxins-13-00552-s001.zip › toxins-1293568-SI.pdf]

Supplementary Materials: Bacterial Enrichment Cultures Biotransform the Mycotoxin Deoxynivalenol into A Novel Metabolite Toxic to Plant and Porcine Cells

Ilse Vanhoutte, Caroline De Tender, Kristel Demeyere, Mohamed F. Abdallah, Sarah Ommeslag, Pieter Vermeir, Sarah De Saeger, Jane Debode, Evelyne Meyer, Siska Croubels, Kris Audenaert and Leen De Gelder

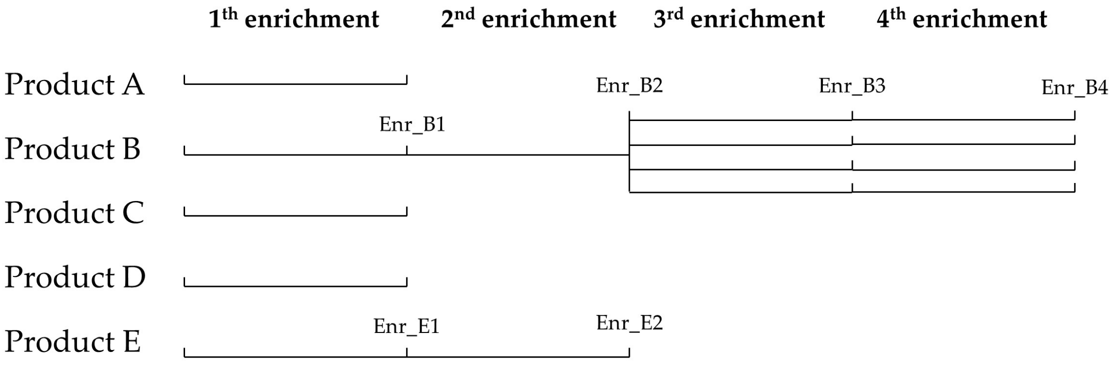

Figure S1. Scheme of experimental setup of enrichment of microbial products A, B, C, D and E. After the first enrichment, two enrichment cultures Enr_B1 and Enr_E1 were obtained capable of degrading DON after 6 weeks.

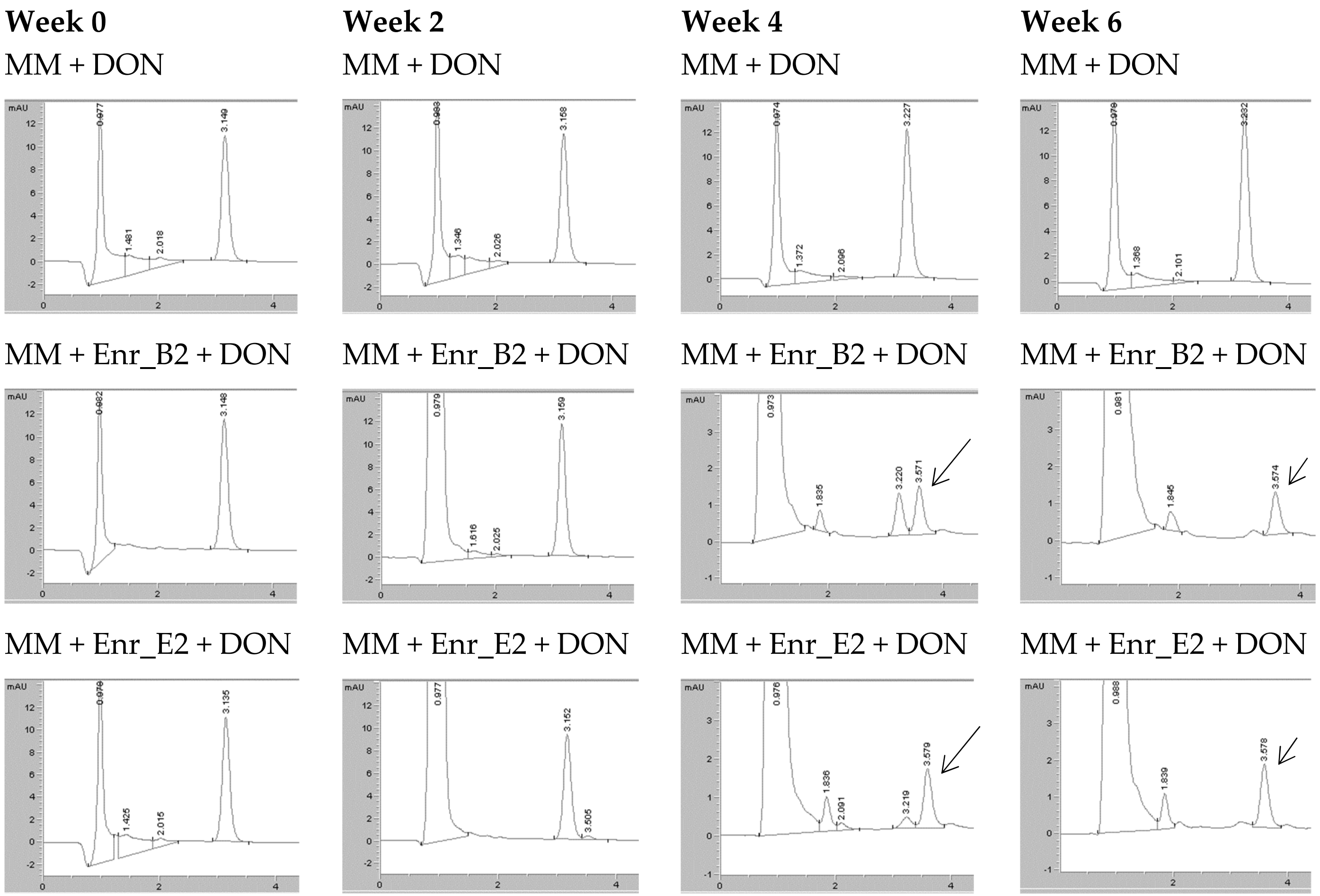

Figure S2. Chromatograms of HPLC-UV analysis at week 0, 2, 4 and 6 of samples treated with enrichment cultures Enr_B2 and Enr_E2. Negative control is included (MM + DON). The absorbance (mAU) is expressed in function of the retention time (r_t). A tentatively observation of a metabolite (r_t = 3.6 min) is made, eluted after DON (r_t = 3.2 min). Method for analysis of DON in MM was validated. Detection of known metabolites of DON with the same method resulted in a different retention time (15-ADON: r_t = 18.2 min, 3-ADON: r_t = 17.4 min, DOM-1: r_t = 7.1 min, DON-3G: r_t = 3.2 min, 3-epi-DON: r_t = 2.5 min).

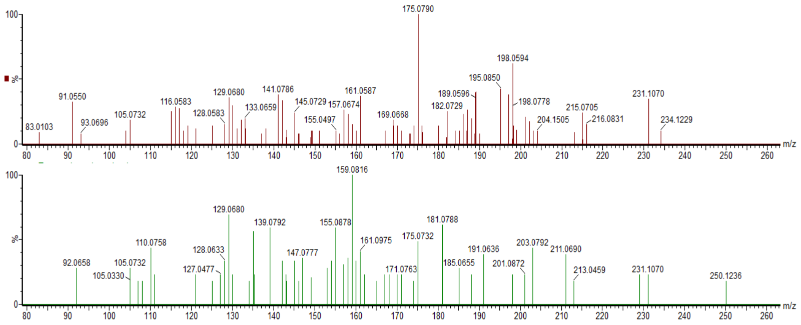

Figure S3. MS/MS spectra of DON (red) and the new metabolite with elemental formula $C_{16}H_{22}O_7$ (green).

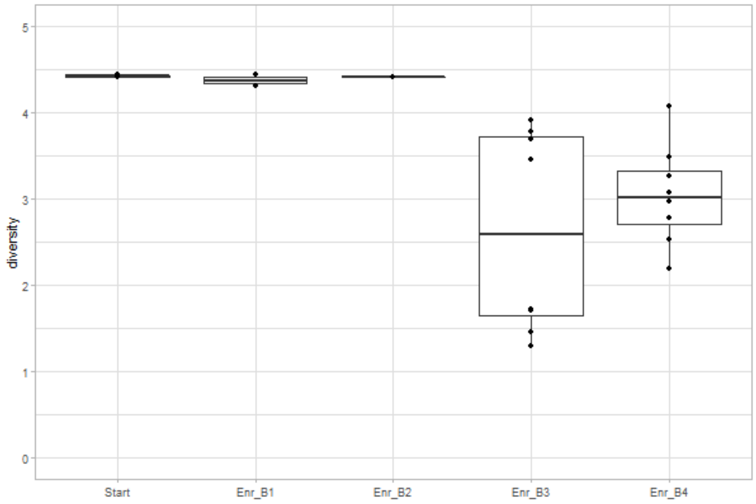

Figure S4. Evaluating the α-diversity: differences between the start product B/1st/2nd enrichment, the 3rd and 4th enrichment in richness versus diversity.

Table S1. Relative abundance of genera per enrichment, starting from product (B), first (Enr_B1), second (Enr_B2), third (Enr_B3) and fourth (Enr_B4) enrichment (only visualization of genera with abundance above 1%).

	Genus	%
Start product B	Other	67.7 ± 0.1
	Hoppeia	7.2 ± 0.5
	Stenotrophobacter	6.0 ± 0.2
	Nitrobacter	5.6 ± 0.7
	Truepera	3.3 ± 0.1
	Lacunisphaera	1.5 ± 0.1
	Altererythrobacter	1.0 ± 0.1
Enr_B1	Other	46.0 ± 0.0
	Acidovorax	12.0 ± 0.4
	Nitrobacter	8.4 ± 0.5
	Mycobacterium	3.8 ± 0.3
	Hydrogenophaga	3.7 ± 0.3
	Thermomonas	3.3 ± 0.1
	Arenibacter	2.8 ± 0.6
	Pseudomonas	2.7 ± 0.9
	Cephaloticoccus	2.5 ± 0.7
	Dokdonella	2.1 ± 0.4
	Qipengyuania	1.8 ± 0.1
	Stenotrophobacter	1.1 ± 0.4
Enr_B2	Other	44.3
	Acidovorax	8.5
	Nitrobacter	7.5
	Thermomonas	5.4
	Arenibacter	4.3
	Mycobacterium	3.9
	Dokdonella	3.3
	Cephaloticoccus	3.1
	Hydrogenophaga	2.8
	Rhodococcus	2.7
	Pseudomonas	1.8
	Hyphomicrobium	1.5
	Pseudonocardia	1.1
	Stenotrophobacter	1.1
	Acinetobacter	1.1
	Phaeodactylibacter	1.1

	Genus	%		Genus	%
Enr_B3_r1+2	*Pseudomonas*	70.6 ± 6.0		*Gordonia*	8.1 ± 3.0
	Other	8.7 ± 2.2		*Pseudarthrobacter*	8.0 ± 0.9
	Sphingopyxis	5.7 ± 1.1		*Terrimonas*	3.9 ± 0.8
	Thermomonas	2.2 ± 0.6		*Shinella*	3.7 ± 0.6
	Dokdonella	2.1 ± 0.2		*Brevibacterium*	3.5 ± 0.7
	Hydrogenophaga	2.0 ± 0.4		*Stenotrophomonas*	3.0 ± 0.2
	Pseudarthrobacter	1.9 ± 0.4		*Ochrobactrum*	2.4 ± 0.1
	Sphingobium	1.5 ± 0.4		*Ferruginibacter*	1.7 ± 0.2
Enr_B3_r3+4	Other	31.9 ± 1.5		*Rhodanobacter*	1.3 ± 0.3
	Thermomonas	16.3 ± 1.1		*Pseudopedobacter*	1.0 ± 0.2
	Pseudomonas	9.6 ± 1.4	Enr_B4_r3	*Sphingopyxis*	33.6 ± 19.5
	Pseudarthrobacter	5.9 ± 0.7		Other	16.2 ± 16.7
	Sphingobium	5.0 ± 0.7		*Ochrobactrum*	10.2 ± 5.7
	Pseudoxanthomonas	3.4 ± 0.8		*Gordonia*	6.8 ± 4.2
	Sphingopyxis	3.3 ± 0.9		*Brevibacterium*	5.4 ± 1.5
	Blastocatella	2.3 ± 0.9		*Rhodococcus*	4.5 ± 6.3
	Rhodanobacter	2.4 ± 0.6		*Pseudoxanthomonas*	3.6 ± 2.3
	Brevibacterium	2.0 ± 0.2		*Acinetobacter*	3.5 ± 4.8
	Sphingomonas	1.5 ± 1.5		*Thermomonas*	3.3 ± 1.9
	Acinetobacter	1.4 ± 0.6		*Massilia*	2.6 ± 3.7
	Dokdonella	1.3 ± 0.8		*Shinella*	1.9 ± 1.0
	Hyphomicrobium	1.3 ± 0.3		*Pseudarthrobacter*	1.6 ± 0.8
	Acidovorax	1.2 ± 0.6	Enr_B4_r4	*Sphingopyxis*	18.7 ± 2.3
	Ochrobactrum	1.1 ± 0.3		Other	17.4 ± 0.4
Enr_B4_r1	*Sphingopyxis*	41.4 ± 4.8		*Pseudoxanthomonas*	16.5 ± 2.4
	Pseudoxanthomonas	12.5 ± 0.4		*Ochrobactrum*	11.8 ± 1.7
	Ochrobactrum	11.6 ± 0.4		*Pseudarthrobacter*	10.9 ± 2.2
	Other	10.5 ± 0.9		*Pseudomonas*	3.3 ± 3.4
	Pseudarthrobacter	6.8 ± 0.4		*Bacillus*	2.5 ± 3.5
	Terrimonas	3.3 ± 0.6		*Rhodanobacter*	2.3 ± 0.2
	Streptomyces	2.7 ± 3.6		*Acinetobacter*	1.8 ± 1.3
	Ferruginibacter	1.9 ± 0.1		*Streptomyces*	1.7 ± 1.5
	Singulisphaera	1.5 ± 0.1		*Hyphomicrobium*	1.6 ± 0.5
	Shinella	1.1 ± 0.2		*Ferruginibacter*	1.5 ± 0.0
Enr_B4_r2	*Sphingopyxis*	20.6 ± 0.4		*Pseudopedobacter*	1.4 ± 0.6
	Pseudoxanthomonas	13.4 ± 1.4		*Sphingomonas*	1.3 ± 0.6
	Other	13.7 ± 0.3		*Shinella*	1.2 ± 0.1
	Thermomonas	12.0 ± 0.6			

Table S2. Difference in community composition between 1st and 3rd enrichment, 3rd and 4th enrichment, and 1st and 4th enrichment (expressed as increase in relative abundance).

	Increase of genera in relative abundance		
	**Enr_B1 vs Enr_B3**	**Enr_B3 vs Enr_B4**	**Enr_B1 vs Enr_B4**
Ochrobactrum	10.4x	4.9x	15.3x
Aeromicrobium	10.1x	*	9.9x
Pseudoxanthomonas	7.6x	3.4x	11.0x
Shinella	4.4x	2.8x	7.2x
Pseudarthrobacter	2.4x	1.5x	3.9x
Flavobacterium	-7.1x	7.0x	*
Sphingopyxis	*	2.9x	5.5x
Terrimonas	*	*	13.5x
Pseudaminobacter	*	*	10.6x

* No increase/decrease observed.

Table S3. Metabolites of DON with corresponding elemental formula, structure, precursor and product ions in positive mode in LC-MS/MS analysis.

**Molecule**	**Elemental formula**	**Structure**	**Precursor ion (m/z) [M+H+]**	**Product ions (m/z) (CE – CV)[1]**	**References**
DON	$C_{15}H_{20}O_6$	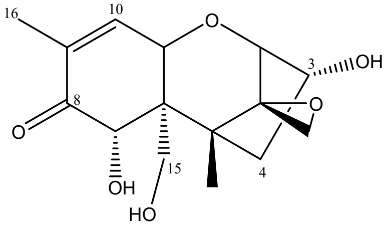	297	279<br>267<br>261<br>249 (9 – 40)<br>231 (9 – 40)	[33-37]
DOM-1	$C_{15}H_{20}O_5$	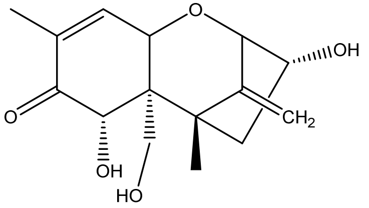	281.1	215.1 (9 – 40)<br>233.1 (9 – 40)	[33,35,38,39]
3-keto-DON	$C_{15}H_{18}O_6$	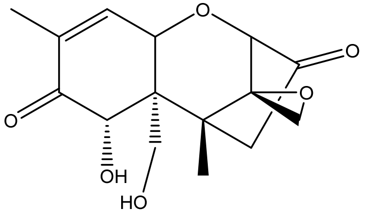	295	247	[11,18,21,35]
3-epi-DON	$C_{15}H_{20}O_6$	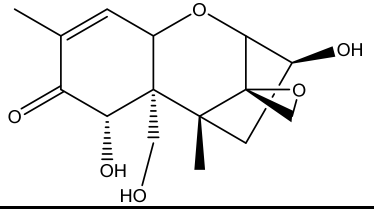	297	279<br>267<br>261<br>249<br>231	[36]

epimer of DOM-1	$C_{15}H_{20}O_5$	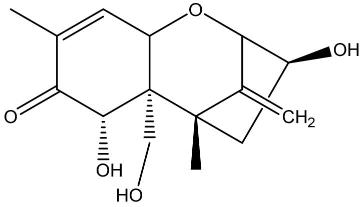	281.1	233.1	[12]
16-HDON	$C_{15}H_{20}O_7$	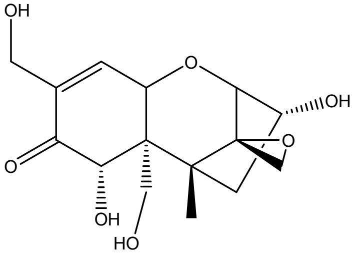	*[M-H$^-$]* 311	*[M-X$^-$]* 293 281 263	[30]
DON + MW(H_2O)	$C_{15}H_{22}O_7$	/	*[M-H$^-$]* 313.4		[29]
DON + MW(CH_2O)	$C_{16}H_{22}O_7$	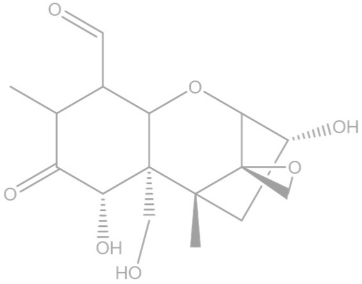 [1]	327.4	309.1 279.1 261.1	
9-hydroxymethyl DON lactone	$C_{16}H_{22}O_7$	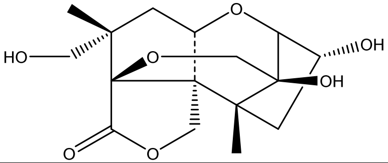	327	309 299 269	[34]

[1]CE = Collision energy (eV) – CV = Cone Voltage (v). [1] Hypothesis: proposed structure of the new metabolite: addition of aldehyde group at C10.

Table S4. Phyto- or cytotoxic assays performed on DON metabolites.

DON metabolite	Phyto- or cytotoxic assay	Conclusion	References
DOM-1	▪ 5-bromo-2'-deoxyuridine (BrdU) incorporation assay assessing DNA-synthesis	IC_{50} = 54 times higher than DON	[40]
	▪ differentiated intestinal porcine epithelial cells (IPEC-J2): lysosomal activity, total protein content, membrane integrity	unaffected at equimolar concentrations in all assays	[41]
	▪ trout gill (RTgill-W1), pig intestinal cells (IPEC-1 and IPEC-J2), human liver cells (HepG2)	no effect on viability	[42]
	▪ human intestinal epithelial cells and pig intestinal explants	not cytotoxic, no change of oxygen consumption, no impairing of barrier function, absence of MAPKinase (mitogen-activated protein kinase) activation	[43]
	▪ intestinal, liver and immune toxicity in sensitive pig model	not toxic in model, but retained some immune-modulatory properties of DON, particularly its ability to stimulate a specific antibody response during a vaccination protocol	[44]
	▪ piglets	non-toxic	[45]
3-epi-DON	▪ bioassay assessing metabolic activity using Caco-2 cells	IC_{50} value = 357 times higher than DON	[46]
	▪ BrdU assay assessing DNA synthesis using 3T3 cells	IC_{50} value = 1181 times higher than DON	[46]
	▪ wheat seedlings	non-toxic	[27]
	▪ B6C3F$_1$ mice during short term oral exposure	at least 50 times less toxic than DON	[46,47]
	▪ human intestinal epithelial cells and pig intestinal explants	not cytotoxic, no change of oxygen consumption, no impairing of barrier function, absence of MAPKinase (mitogen-activated protein kinase) activation	[43]

	▪ piglets	non-toxic	[45]
3-keto-DON	▪ bioassay based on the mitogen-induced and mitogen-free profilerations of mouse spleen lymphocytes	remarkably decreased (to less than one tenth) immunosuppressive toxicity relative to DON	[18]
	▪ bioassay assessing metabolic activity using Caco-2 cells	IC_{50} value = 3 times higher than DON	[46]
	▪ BrdU assay assessing DNA synthesis using 3T3 cells	IC_{50} value = 5 times higher than DON	[46]
	▪ wheat seedlings	non-toxic	[27]
epimer of DOM-1	▪ bioassay using aquatic plant *Lemna minor* L.	first indication of non-phytotoxic effect (diluted)	[12]
	▪ cytotoxic assay using IPEC-J2 cells	first indication of non-cytotoxic effect (diluted)	
16H-DON	▪ wheat seedlings	dramatically reduction of toxicity	[30]
DON + MW(CH_2O)	▪ bioassay using aquatic plant *Lemna minor* L.	first indication of strongly phytotoxic effect (diluted)	
	▪ cytotoxic assay using IPEC-J2 cells	first indication of cytotoxic effect (diluted)	

Table S5. Validation parameters for DON in minimal medium (HPLC-UV analysis).

LOD (mg/L)	LOQ (mg/L)	Spiked level (mg/L)	AR (%)	Rep (%)	IP (%)
0.21	0.35	0.35	102.1	8.2	12.5
		1	99.6	3.1	5.4
		2	99.4	3.2	3.3
		5	99.5	1.7	1.6
		10	98.8	1.6	2.0

LOD = limit of detection; LOQ = limit of quantification; AR= apparent recovery; Rep= repeatability (σ^2_{within}; intraday precision); IP = intermediate precision (σ^2_{total}; interday precision).

References

1. Guan, S.; He, J.W.; Young, J.C.; Zhu, H.H.; Li, X.Z.; Ji, C.; Zhou, T. Transformation of trichothecene mycotoxins by microorganisms from fish digesta. *Aquaculture* **2009**, *290*, 290-295, doi:10.1016/j.aquaculture.2009.02.037.
2. Islam, R.; Zhou, T.; Young, J.C.; Goodwin, P.H.; Pauls, K.P. Aerobic and anaerobic de-epoxydation of mycotoxin deoxynivalenol by bacteria originating from agricultural soil. *World J Microb Biot* **2012**, *28*, 7-13, doi:10.1007/s11274-011-0785-4.
3. Ahad, R.; Zhou, T.; Lepp, D.; Pauls, K.P. Microbial detoxification of eleven food and feed contaminating trichothecene mycotoxins. *Bmc Biotechnol* **2017**, *17*, doi:ARTN 30

10.1186/s12896-017-0352-7.

4. He, W.J.; Yuan, Q.S.; Zhang, Y.B.; Guo, M.W.; Gong, A.D.; Zhang, J.B.; Wu, A.B.; Huang, T.; Qu, B.; Li, H.P., et al. Aerobic De-Epoxydation of Trichothecene Mycotoxins by a Soil Bacterial Consortium Isolated Using In Situ Soil Enrichment. *Toxins* **2016**, *8*, doi:ARTN 277

10.3390/toxins8100277.

5. Fuchs, E.; Binder, E.M.; Heidler, D.; Krska, R. Structural characterization of metabolites after the microbial degradation of type A trichothecenes by the bacterial strain BBSH 797. *Food Addit Contam* **2002**, *19*, 379-386, doi:10.1080/02652030110091154.
6. Binder, E.M.; Binder, J. Strain of Eubacterium that Detoxifies Trichothecenes. 2004.
7. Yu, H.; Zhou, T.; Gong, J.H.; Young, C.; Su, X.J.; Li, X.Z.; Zhu, H.H.; Tsao, R.; Yang, R. Isolation of deoxynivalenol-transforming bacteria from the chicken intestines using the approach of PCR-DGGE guided microbial selection. *Bmc Microbiol* **2010**, *10*, doi:Artn 182

10.1186/1471-2180-10-182.

8. Gao, X.J.; Mu, P.Q.; Wen, J.K.; Sun, Y.; Chen, Q.M.; Deng, Y.Q. Detoxification of trichothecene mycotoxins by a novel bacterium, Eggerthella sp DII-9. *Food Chem Toxicol* **2018**, *112*, 310-319, doi:10.1016/j.fct.2017.12.066.
9. Gao, X.J.; Mu, P.Q.; Zhu, X.H.; Chen, X.X.; Tang, S.L.; Wu, Y.T.; Miao, X.; Wang, X.H.; Wen, J.K.; Deng, Y.Q. Dual Function of a Novel Bacterium, Slackia sp. D-G6: Detoxifying Deoxynivalenol and Producing the Natural Estrogen Analogue, Equol. *Toxins* **2020**, *12*, doi:ARTN 85

10.3390/toxins12020085.

10. Islam, R. Isolation, Characterization and Genome Sequencing of a Soil-Borne Citrobacter Freundii Strain Capable of Detoxifying Trichothecene Mycotoxins. Guelph, U.o., Ed. Guelph, ON, Canada, 2012.
11. Volkl, A.; Vogler, B.; Schollenberger, M.; Karlovsky, P. Microbial detoxification of mycotoxin deoxynivalenol. *J Basic Microb* **2004**, *44*, 147-156, doi:10.1002/jobm.200310353.
12. Vanhoutte, I.; De Mets, L.; De Boevre, M.; Uka, V.; Di Mavungu, J.D.; De Saeger, S.; De Gelder, L.; Audenaert, K. Microbial Detoxification of Deoxynivalenol (DON), Assessed via a Lemna minor L. Bioassay, through Biotransformation to 3-epi-DON and 3-epi-DOM-1. *Toxins* **2017**, *9*, doi:ARTN 63

10.3390/toxins9020063.

13. Wilson, N.M.; McMaster, N.; Gantulga, D.; Soyars, C.; McCormick, S.P.; Knott, K.; Senger, R.S.; Schmale, D.G. Modification of the Mycotoxin Deoxynivalenol Using Microorganisms Isolated from Environmental Samples. *Toxins* **2017**, *9*, doi:ARTN 141

10.3390/toxins9040141.

14. Wang, G.; Wang, Y.X.; Man, H.Z.; Lee, Y.W.; Shi, J.R.; Xu, J.H. Metabolomics-guided analysis reveals a two-step epimerization of deoxynivalenol catalyzed by the bacterial consortium IFSN-C1. *Appl Microbiol Biot* **2020**, *104*, 6045-6056, doi:10.1007/s00253-020-10673-1.
15. Zhai, Y.Y.; Zhong, L.; Gao, H.; Lu, Z.X.; Bie, X.M.; Zhao, H.Z.; Zhang, C.; Lu, F.X. Detoxification of Deoxynivalenol by a Mixed Culture of Soil Bacteria With 3-epi-Deoxynivalenol as the Main Intermediate. *Front Microbiol* **2019**, *10*, doi:ARTN 2172

10.3389/fmicb.2019.02172.

16. Zhang, J.; Qin, X.J.; Guo, Y.P.; Zhang, Q.Q.; Ma, Q.G.; Ji, C.; Zhao, L.H. Enzymatic degradation of deoxynivalenol by a novel bacterium, Pelagibacterium halotolerans ANSP101. *Food Chem Toxicol* **2020**, *140*, doi:ARTN 111276

10.1016/j.fct.2020.111276.

17. Sato, I.; Ito, M.; Ishizaka, M.; Ikunaga, Y.; Sato, Y.; Yoshida, S.; Koitabashi, M.; Tsushima, S. Thirteen novel deoxynivalenol-degrading bacteria are classified within two genera with distinct degradation mechanisms. *Fems Microbiol Lett* **2012**, *327*, 110-117, doi:10.1111/j.1574-6968.2011.02461.x.

18. Shima, J.; Takase, S.; Takahashi, Y.; Iwai, Y.; Fujimoto, H.; Yamazaki, M.; Ochi, K. Novel detoxification of the trichothecene mycotoxin deoxynivalenol by a soil bacterium isolated by enrichment culture. *Appl Environ Microb* **1997**, *63*, 3825-3830, doi:Doi 10.1128/Aem.63.10.3825-3830.1997.
19. Carere, J.; Hassan, Y.I.; Lepp, D.; Zhou, T. The enzymatic detoxification of the mycotoxin deoxynivalenol: identification of DepA from the DON epimerization pathway. *Microb Biotechnol* **2018**, *11*, 1106-1111, doi:10.1111/1751-7915.12874.
20. Carere, J.; Hassan, Y.I.; Lepp, D.; Zhou, T. The Identification of DepB: An Enzyme Responsible for the Final Detoxification Step in the Deoxynivalenol Epimerization Pathway in Devosia mutans 17-2-E-8. *Front Microbiol* **2018**, *9*, doi:ARTN 1573
10.3389/fmicb.2018.01573.
21. Hassan, Y.I.; He, J.W.; Perilla, N.; Tang, K.J.; Karlovsky, P.; Zhou, T. The enzymatic epimerization of deoxynivalenol by Devosia mutans proceeds through the formation of 3-keto-DON intermediate. *Sci Rep-Uk* **2017**, *7*, doi:ARTN 6929
10.1038/s41598-017-07319-0.
22. Hassan, Y.I.; Lepp, D.; He, J.; Zhou, T. Draft Genome Sequences of Devosia sp. Strain 17-2-E-8 and Devosia riboflavina Strain IFO13584. *Genome announcements* **2014**, *2*, doi:10.1128/genomeA.00994-14.
23. He, J.W. Detoxification of Deoxynivalenol by a Soil Bacterium Devosia mutans 17-2-E-8. Guelph, U.o., Ed. Guelph, ON, Canada, 2015.
24. He, W.J.; Shi, M.M.; Yang, P.; Huang, T.; Zhao, Y.; Wu, A.B.; Dong, W.B.; Li, H.P.; Zhang, J.B.; Liao, Y.C. A quinone-dependent dehydrogenase and two NADPH-dependent aldo/keto reductases detoxify deoxynivalenol in wheat via epimerization in a Devosia strain. *Food Chem* **2020**, *321*, doi:ARTN 126703
10.1016/j.foodchem.2020.126703.
25. Wang, G.; Wang, Y.X.; Ji, F.; Xu, L.M.; Yu, M.Z.; Shi, J.R.; Xu, J.H. Biodegradation of deoxynivalenol and its derivatives by Devosia insulae A16. *Food Chem* **2019**, *276*, 436-442, doi:10.1016/j.foodchem.2018.10.011.
26. Wang, Y.; Zhang, H.H.; Zhao, C.; Han, Y.T.; Liu, Y.C.; Zhang, X.L. Isolation and characterization of a novel deoxynivalenol-transforming strain Paradevosia shaoguanensis DDB001 from wheat field soil. *Letters in applied microbiology* **2017**, *65*, 414-422, doi:10.1111/lam.12790.
27. He, W.J.; Zhang, L.M.; Yi, S.Y.; Tang, X.L.; Yuan, Q.S.; Guo, M.W.; Wu, A.B.; Qu, B.; Li, H.P.; Liao, Y.C. An aldo-keto reductase is responsible for Fusarium toxin-degrading activity in a soil Sphingomonas strain. *Sci Rep-Uk* **2017**, *7*, doi:ARTN 9549
10.1038/s41598-017-08799-w.
28. Ikunaga, Y.; Sato, I.; Grond, S.; Numaziri, N.; Yoshida, S.; Yamaya, H.; Hiradate, S.; Hasegawa, M.; Toshima, H.; Koitabashi, M., et al. Nocardioides sp. strain WSN05-2, isolated from a wheat field, degrades deoxynivalenol, producing the novel intermediate 3-epi-deoxynivalenol. *Appl Microbiol Biot* **2011**, *89*, 419-427, doi:10.1007/s00253-010-2857-z.
29. He, C.H.; Fan, Y.H.; Liu, G.F.; Zhang, H.B. Isolation and Identification of a Strain of Aspergillus Tubingensis With Deoxynivalenol Biotransformation Capability. *Int J Mol Sci* **2008**, *9*, 2366-2375, doi:10.3390/ijms9122366.
30. Ito, M.; Sato, I.; Ishizaka, M.; Yoshida, S.; Koitabashi, M.; Yoshida, S.; Tsushima, S. Bacterial Cytochrome P450 System Catabolizing the Fusarium Toxin Deoxynivalenol. *Appl Environ Microb* **2013**, *79*, 1619-1628, doi:10.1128/Aem.03227-12.
31. Ito, M.; Sato, I.; Koitabashi, M.; Yoshida, S.; Imai, M.; Tsushima, S. A novel actinomycete derived from wheat heads degrades deoxynivalenol in the grain of wheat and barley affected by Fusarium head blight. *Appl Microbiol Biot* **2012**, *96*, 1059-1070, doi:10.1007/s00253-012-3922-6.
32. Wang, S.W.; Hou, Q.Q.; Guo, Q.Q.; Zhang, J.; Sun, Y.M.; Wei, H.; Shen, L.X. Isolation and Characterization of a Deoxynivalenol-Degrading Bacterium Bacillus licheniformis YB9 with the Capability of Modulating Intestinal Microbial Flora of Mice. *Toxins* **2020**, *12*, doi:ARTN 184
10.3390/toxins12030184.
33. Awad, W.A.; Ghareeb, K.; Bohm, J.; Zentek, J. Decontamination and detoxification strategies for the Fusarium mycotoxin deoxynivalenol in animal feed and the effectiveness of microbial biodegradation. *Food Addit Contam A* **2010**, *27*, 510-520, doi:10.1080/19440040903571747.
34. Bretz, M.; Beyer, M.; Cramer, B.; Knecht, A.; Humpf, H.U. Thermal degradation of the Fusarium mycotoxin deoxynivalenol. *J Agr Food Chem* **2006**, *54*, 6445-6451, doi:10.1021/jf061008g.
35. Hassan, Y.I.; Zhou, T. Addressing the mycotoxin deoxynivalenol contamination with soil-derived bacterial and enzymatic transformations targeting the C3 carbon. *World Mycotoxin J* **2018**, *11*, 101-111, doi:10.3920/Wmj2017.2259.

36. He, J.W.; Yang, R.; Zhou, T.; Boland, G.J.; Scott, P.M.; Bondy, G.S. An epimer of deoxynivalenol: purification and structure identification of 3-epi-deoxynivalenol. *Food Addit Contam A* **2015**, *32*, 1523-1530, doi:10.1080/19440049.2015.1072771.
37. Sobrova, P.; Adam, V.; Vasatkova, A.; Beklova, M.; Zeman, L.; Kizek, R. Deoxynivalenol and its toxicity. *Interdisciplinary toxicology* **2010**, *3*, 94-99, doi:10.2478/v10102-010-0019-x.
38. Yoshizawa, T.; Takeda, H.; Ohi, T. Structure of a Novel Metabolite from Deoxynivalenol, a Trichothecene Myco-Toxin, in Animals. *Agr Biol Chem Tokyo* **1983**, *47*, 2133-2135, doi:Doi 10.1080/00021369.1983.10865926.
39. Zhu, Y.; Hassan, Y.I.; Shao, S.; Zhou, T. Employing immuno-affinity for the analysis of various microbial metabolites of the mycotoxin deoxynivalenol. *J Chromatogr A* **2018**, *1556*, 81-87, doi:10.1016/j.chroma.2018.04.067.
40. Eriksen, G.S.; Pettersson, H.; Lundh, T. Comparative cytotoxicity of deoxynivalenol, nivalenol, their acetylated derivatives and de-epoxy metabolites. *Food Chem Toxicol* **2004**, *42*, 619-624, doi:10.1016/j.fct.2003.11.006.
41. Springler, A.; Hessenberger, S.; Reisinger, N.; Kern, C.; Nagl, V.; Schatzmayr, G.; Mayer, E. Deoxynivalenol and its metabolite deepoxy-deoxynivalenol: multi-parameter analysis for the evaluation of cytotoxicity and cellular effects. *Mycotoxin Res* **2017**, *33*, 25-37, doi:10.1007/s12550-016-0260-z.
42. Mayer, E.; Novak, B.; Springler, A.; Schwartz-Zimmermann, H.E.; Nagl, V.; Reisinger, N.; Hessenberger, S.; Schatzmayr, G. Effects of deoxynivalenol (DON) and its microbial biotransformation product deepoxy-deoxynivalenol (DOM-1) on a trout, pig, mouse, and human cell line. *Mycotoxin Res* **2017**, *33*, 297-308, doi:10.1007/s12550-017-0289-7.
43. Pierron, A.; Mimoun, S.; Murate, L.S.; Loiseau, N.; Lippi, Y.; Bracarense, A.P.F.L.; Schatzmayr, G.; He, J.W.; Zhou, T.; Moll, W.D., et al. Microbial biotransformation of DON: molecular basis for reduced toxicity. *Sci Rep-Uk* **2016**, *6*, doi:ARTN 29105
10.1038/srep29105.
44. Pierron, A.; Bracarense, A.P.F.L.; Cossalter, A.M.; Laffitte, J.; Schwartz-Zimmermann, H.E.; Schatzmayr, G.; Pinton, P.; Moll, W.D.; Oswald, I.P. Deepoxy-deoxynivalenol retains some immune-modulatory properties of the parent molecule deoxynivalenol in piglets. *Arch Toxicol* **2018**, *92*, 3381-3389, doi:10.1007/s00204-018-2293-x.
45. Bracarense, A.P.F.L.; Pierron, A.; Pinton, P.; Gerez, J.R.; Schatzmayr, G.; Moll, W.D.; Zhou, T.; Oswald, I.P. Reduced toxicity of 3-epi-deoxynivalenol and de-epoxy-deoxynivalenol through deoxynivalenol bacterial biotransformation: In vivo analysis in piglets. *Food Chem Toxicol* **2020**, *140*, doi:ARTN 111241
10.1016/j.fct.2020.111241.
46. He, J.W.; Bondy, G.S.; Zhou, T.; Caldwell, D.; Boland, G.J.; Scott, P.M. Toxicology of 3-epi-deoxynivalenol, a deoxynivalenol-transformation product by Devosia mutans 17-2-E-8. *Food Chem Toxicol* **2015**, *84*, 250-259, doi:10.1016/j.fct.2015.09.003.
47. Zhou, T.; He, J. Bacterial Isolate, Methods of Isolating Bacterial Isolates and Methods for Detoxification of Trichothecene Mycotoxins. 6 October 2010, 2010.
